# Identification of Compound Heterozygous *DPM1* Variants in a Pediatric Patient With Congenital Disorder of Glycosylation Type Ie

**DOI:** 10.1155/crpe/5573260

**Published:** 2025-11-19

**Authors:** Wei Song, Wenzhi Zhou, Liu Yang, Li Tang

**Affiliations:** ^1^Department of Children Rehabilitation, Chengdu Women's and Children's Central Hospital, School of Medicine, University of Electronic Science and Technology of China, Chengdu, Sichuan, China; ^2^Department of Healthcare, Chengdu Women's and Children's Central Hospital, School of Medicine, University of Electronic Science and Technology of China, Chengdu, Sichuan, China

**Keywords:** congenital disorder of glycosylation, *DPM1* gene, genetic metabolic disease

## Abstract

Congenital disorders of glycosylation (CDG) constitute a group of rare genetic metabolic diseases caused by defects in the synthesis and modification of oligosaccharides. CDG-Ie is a rare subtype caused by mutations in the *DPM1* gene. We describe a female patient who presented with ocular abnormalities, motor retardation, hypotonia, hepatic dysfunction, elevated creatine kinase, abnormal electroencephalogram (EEG), and abnormal cranial magnetic resonance imaging (MRI) findings, which have been reported in previous cases. However, we note that this case had previously undescribed findings of the CDG-Ie phenotype, including hearing abnormalities and decreased parathyroid hormone levels. She carries compound heterozygous variants in the *DPM1* gene: a paternal [c.1A>G (p.M1V)] start codon mutation and a maternal [c.371A>G (p.H124R), (NM_003859)] missense variant. We present the 12th case known worldwide to date.

## 1. Introduction

Congenital disorder of glycosylation (CDG) are a group of rare genetic metabolic disorders caused by defects in glycoprotein synthesis. This large class of monogenic diseases stems from impairments in the synthesis and modification of oligosaccharides. The majority of cases are inherited in an autosomal recessive manner, while a minority are inherited in an autosomal dominant or X-linked manner.

Glycosylation is an important process in eukaryotic cells where sugar chains are attached to proteins and lipids, playing a crucial role in the biological functions of many proteins. It involves a series of steps, including transport, catalysis, linking, and trimming. Insufficient or erroneous glycosylation can lead to abnormal protein function and disease [1]. There are four main types of defects [2]: Type I involves defects in N-linked glycosylation, which is a post-translational modification of proteins that occurs in the endoplasmic reticulum and Golgi apparatus, involving the assembly of carbohydrate chains, covalent linkage with asparagine residues, and trimming—hence the term N-linked [1]; Type II involves defects in O-linked glycosylation, which refers to the attachment of sugar chains, such as mannose, to the hydroxyl groups of serine or threonine residues; Type III involves combined defects in N- and O-linked glycosylation; Type IV involves defects in lipid and glycosylphosphatidylinositol (GPI) anchoring. Glycosylation defects can affect multiple systems and organs, presenting with diverse clinical manifestations, often leading to misdiagnosis or underdiagnosis.

Approximately 160 types of CDG have been reported, each defined and named according to a specific gene. Among them, CDG-Ie (OMIM: 608799) is a rare type, first reported in 2000 [3, 4], with only 11 cases reported worldwide to date [1, 3–9]. Its rarity is primarily due to defects in the *DPM1* gene, which lead to reduced activity of dolichol-phosphate mannosyltransferase 1 (DPM1) and impaired synthesis of dolichol-phosphate-mannose (DPM) complexes [10]. This leads to combined defects in both N-linked and O-linked glycosylation [11]. The *DPM1* gene is most localized in the endoplasmic reticulum within the cell. However, mRNA expression studies show that it is expressed throughout the body, particularly in the muscle and nervous system. This article presents the clinical manifestations and genetic test results of a CDG-Ie case diagnosed at our hospital.

## 2. Case Presentation

The patient is a girl, G1P1, born at 39^+2^ weeks of gestation with a birth weight of 3670 g. The parents are nonconsanguineous, and there were no abnormalities during the mother's pregnancy. Due to suspected intrauterine distress, the patient underwent a cesarean section with meconium-stained amniotic fluid and Apgar scores of 4-8-8 (at 1-5-10 min after birth). After delivery, the patient exhibited tachypnea, grunting, cyanosis, and poor response upon admission. Blood glucose was measured at 1.71 mmol/L. Laboratory results included aspartate aminotransferase (AST) 45 U/L, alanine aminotransferase (ALT) 142 U/L, total bilirubin 52.9 μmol/L, direct bilirubin 15.0 μmol/L, and indirect bilirubin 37.9 μmol/L. In addition, glutamate dehydrogenase was 105 U/L, lactate dehydrogenase (LDH) 2809 U/L, creatine kinase (CK) 8971 U/L, and creatine kinase isoenzyme (CK-MB) 389 U/L. A chest X-ray showed patchy and nodular densities in both lower lung fields. Brain magnetic resonance imaging (MRI) revealed no abnormalities. On the 4th day postpartum, laboratory results showed AST 53 U/L, alpha-hydroxybutyrate dehydrogenase 566 U/L, LDH 609 U/L, CK 2098 U/L, and CK-MB 131 U/L. After 5 days of treatment with antibiotics, vitamin K administration for bleeding prevention, and nutritional support, the patient's clinical condition improved, and she was discharged.

At 2 months of age, during a routine child health check-up, the patient was referred due to low muscle tone. Physical examination revealed no specific facial features, mild nystagmus in both eyes, limited pursuit movement, poor head control, decreased muscle tone in both upper limbs, and increased deep tendon reflexes. Laboratory results showed AST 94.5 U/L, ALT 80.1 U/L, alpha-hydroxybutyrate dehydrogenase 336.3 U/L, LDH 408.9 U/L, CK 735 U/L, and globulin 8.4 g/L. Parathyroid hormone level was 4.2 pg/mL. Lymphocyte values in the hemogram were normal, and no immunodeficiency findings were detected. Blood glucose, electrolytes, renal function, thyroid function, growth hormone, vitamin B12, folate, and vitamin D were all normal. As a result of the Gesell developmental assessment, the patient was diagnosed with global developmental delay. (Development Quotient (DQ) scores: 84 in adaptive skills, 56 in gross motor skills, 82 in fine motor skills, 82 in language skills, and 57 in personal-social skills.) Abdominal and urinary ultrasound showed no significant abnormalities. Video electroencephalogram (EEG) revealed diffuse sharp and slow waves in the bilateral frontal region during both awake and sleep states, predominantly on the left side. Auditory brainstem response (ABR) testing showed elevated hearing thresholds (55 dB) and prolonged absolute latencies of waves I and III bilaterally. Visual evoked potentials (VEPs) were normal. Wrist bone age was appropriate for the patient's age. Brain MRI showed heterogeneous signals in the bilateral basal ganglia, with slightly increased T1-weighted (T1W1) signal in the posterior part of the left caudate nucleus. Bilateral lateral ventricles were full, and the lateral spaces of the bilateral frontal and temporal lobes were widened. The patient was referred to the Beijing Fuyulonghui Genetic Disease Clinic for blood and urine metabolic screening.

Amino acid analysis revealed significantly decreased arginine (1.18 μmol/L) and reduced ornithine (47.85 μmol/L), indicating potential nutritional deficiencies. The free carnitine and acylcarnitine profile showed decreased levels of various acylcarnitines, suggesting nutritional deficiencies. Urinary organic acid analysis did not show typical organic acid metabolism disorder, but elevated concentrations of oxalic acid, succinic acid, glyceric acid, 3-methylglutaconic acid, butyrylglycine, and 3-hydroxyglutaric acid were noted. As a result, no significant pathology was identified other than mild metabolic abnormalities associated with nutritional deficiency. However, further metabolic investigations could not be performed due to the patient's death. Growth hormone treatment was initiated based on a preliminary diagnosis of Prader–Willi syndrome and continued for 2 months.

At 6 months of age, during a follow-up visit, the patient did not present with feeding difficulties, recurrent infections, bleeding tendencies, or seizures but still exhibited poor head control, delayed motor development, low muscle tone, brisk deep tendon reflexes, vowel production, communicative vocalizations, brief attention to toys, and brief visual tracking of faces. At around 7 months of age, the patient developed pneumonia and subsequently died after the parents opted for palliative care.

**Figure 1:**
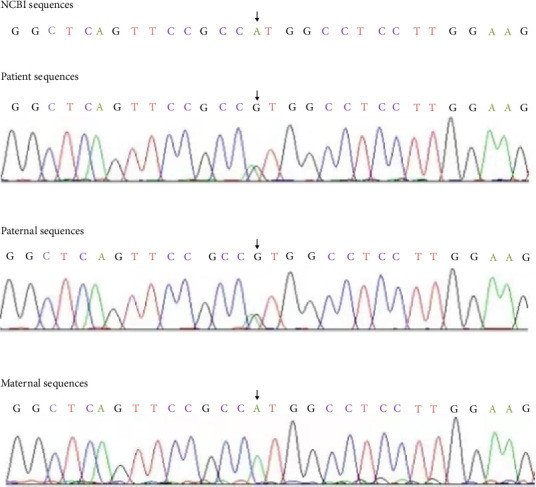
c.1A>G paternal inherited.

**Figure 2:**
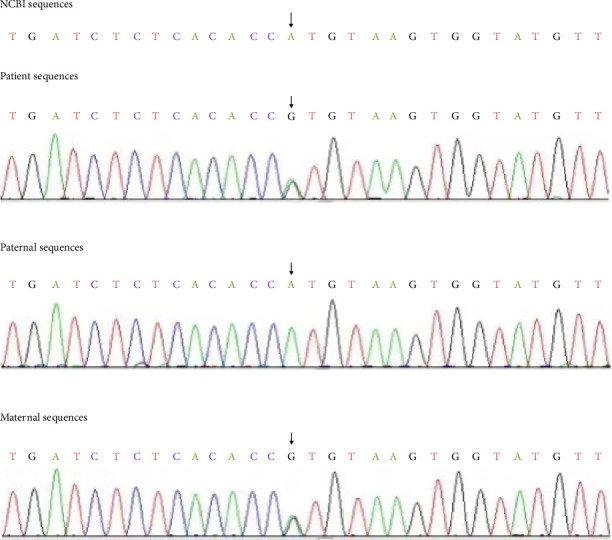
c.371A>G maternal inherited.

After obtaining approval from the medical ethics review board (the Ethics Committee of Chengdu Women's and Children's Hospital, approval number: 202141) and informed parental consent, comprehensive genetic testing was performed. A blood sample was sent to the Beijing Fuyulonghui Genetic Disease Clinic for whole-exome sequencing of single-gene diseases. Chromosomal microarray analysis revealed no abnormalities. The whole exome sequencing results were subsequently validated through PCR–Sanger sequencing at the Genetic and Prenatal Diagnosis Center of the First Affiliated Hospital of Zhengzhou University. As shown in Figures 1 and 2, the genetic analysis revealed compound heterozygous mutations in the DPM1 gene: c.1A>G (p.M1V) and c.371A>G (p.H124R), which are associated with CDG-Ie. Both parents were carriers of these mutations but exhibited normal clinical phenotypes. NM_003859.3(DPM1):c.1A>G mutation in ClinVar RCV001205494.13 previously reported as pathogenic. The c.1A>G (p.M1V) mutation, inherited from the father, is a mutation in the start codon. The rs number is rs139624629, and the frequency of this variant in the population, as recorded in the GnomAD database, is 0.0001029. According to the American College of Medical Genetics and Genomics (ACMG) variant classification guidelines, this mutation is rated as likely pathogenic.

The c.371A>G (p.H124R) variant change, inherited from the mother, is a missense variant change in which the 371st base of the coding region changes from A to G, resulting in the substitution of histidine with arginine at position 124. The rs number is rs2123115725, and the population frequency of this variant change in the gnomAD database is 6.198 − *e*7. According to the ACMG variant classification guidelines, this mutation is also categorized as likely pathogenic (PM2_Supporting + PP3). This variant change has been previously reported in the literature; however, it has not been functionally or clinically correlated with disease to date.

The identified mutations were confirmed through PCR–Sanger sequencing (Figures 1 and 2). Due to the early death of the patient, we were unable to perform biochemical markers such as isoelectric focusing.

## 3. Discussion

The pathogenesis of CDG is not fully understood, but it is generally hypothesized that variations in glycosylation-related genes disrupt the normal glycosylation process, leading to insufficient or incorrect glycosylation of target molecules. This disruption in glycosylation can cause dysfunction or mislocalization of glycosylated compounds, resulting in a range of functional consequences. The overall prevalence and incidence of CDG remain undetermined [12], and no statistics are currently available on its incidence rate in China. However, based on carrier frequencies of pathogenic variations in 53 known genes associated with CDG, the estimated prevalence of CDG in Caucasian and African American populations in the United States is approximately 1 in 10,000 [12].

The most common form of CDG, CDG-Ia, is caused by pathogenic variations in the *PMM2* gene, with an estimated incidence of 1 in 20,000 in Dutch and Danish populations [13]. Most other types of CDG have fewer than 100 reported cases worldwide [14, 15]. In a European survey of 1350 confirmed CDG cases, CDG-Ia represented 61.7% of cases, followed by CDG-Ic at approximately 7.4%, CDG-Iq at 3.1%, CDG-Ik at 3.0%, and CDG-Ib at 2.6%. Notably, CDG-Ie was not reported in this survey [16]. According to Orphanet (https://www.orpha.net/), the prevalence of CDG-Ie is estimated to be less than 1/1,000,000.

CDG-Ie is a rare type of CDG, as reported in the literature. Recent research has identified that the DPM complex, essential for glycosylation, consists of three protein subunits (DPM1, DPM2, and DPM3). The *DPM1* gene, located on chromosome 20q13.13, comprises 10 exons and serves as the cytoplasmic catalytic subunit. It is anchored to the endoplasmic reticulum membrane by DPM3, with DPM2 stabilizing the complex. All three subunits are indispensable for proper DPM transferase function [17]. The pathogenesis of CDG-Ie is primarily due to defects in the *DPM1* gene, resulting in decreased DPM transferase activity. This leads to impaired synthesis of DPM from dolichol-phosphate and GDP-mannose, disrupting various glycosylation processes, including N-glycosylation, O-glycosylation, C-mannosylation, and GPI anchoring, due to the lack of donor substrates. CDG-Ie is inherited in an autosomal recessive manner. In this case, the patient carries a previously unreported variant in the *DPM1* gene, thereby expanding the genetic spectrum of CDG-Ie.

Alpha-dystroglycan (α-DG) is a highly glycosylated protein that links the extracellular matrix to the intracellular cytoskeleton, contributing to cell stability and integrity. Located on the peripheral membrane, α-DG is found in peripheral nerves, skeletal muscles, and brain tissues. Proper glycosylation of α-DG is essential for maintaining muscle cell membrane integrity. Mutations in the *DPM1* gene disrupt the O-glycosylation process of α-DG, resulting in a fragile muscle cell membrane, which can lead to elevated serum creatine kinase levels. This glycosylation defect is associated with α-DGP, characterized by progressive muscle weakness and atrophy [11, 18, 19]. We believe that the elevated creatine kinase and hypotonia in our patient are related to this condition.

Although the genetic mutation sites in this case differ from those previously reported, the clinical features observed, such as ocular abnormalities, delayed motor development, low muscle tone, abnormal liver function, elevated creatine kinase, abnormal EEG, and atypical brain MRI, are consistent with those documented in prior cases [8]. However, this patient also exhibited unique features, including hearing abnormalities, decreased parathyroid hormone levels, significantly reduced arginine, and low ornithine levels, which have not been previously reported in CDG-Ie. It remains unclear if these manifestations are characteristic of CDG-Ie. Although not previously mentioned in the literature, we believe that these differences are related to mRNA expression studies, which show that it is widely expressed in the body. The patient passed away at 7 months of age. The presence of auditory abnormalities and transient visual function disturbances suggests a heightened risk for language, cognitive, and social impairments.

Treatment for CDG-Ie is primarily focused on symptomatic management. Given the involvement of multiple organ systems, long-term, individualized care is necessary and often requires a multidisciplinary approach.

CDG shares clinical features with other hereditary multisystem disorders, such as mitochondrial diseases, peroxisomal disorders, and lysosomal storage diseases. Before genetic testing, this patient was also suspected of having Prader–Willi syndrome and receiving growth hormone therapy, though with limited success. In particular, for patients with severe neurological symptoms who are in neonatal intensive care, we recommend rapid whole exome sequencing analysis, which differs from the traditional approach, and evaluation of patients based on reverse phenotyping according to the results [20]. Families who have been diagnosed are informed about possible genetic risks for their future children during family planning, helping them make informed decisions.

In summary, CDG-Ie is a rare subtype of CDG, with only a small number of reported cases. Genetic testing is instrumental in achieving a definitive diagnosis, enabling more precise management and counseling for affected families.

## Data Availability

The data that support the findings of this study are available on request from the corresponding author. The data are not publicly available due to privacy or ethical restrictions.
